# Revealing the Viable Microbial Community of Biofilm in a Sewage Treatment System Using Propidium Monoazide Combined with Real-Time PCR and Metagenomics

**DOI:** 10.3390/microorganisms12081508

**Published:** 2024-07-23

**Authors:** Jiayin Liang, Xiangqun Zheng, Tianyang Ning, Jiarui Wang, Xiaocheng Wei, Lu Tan, Feng Shen

**Affiliations:** 1 Agro-Environmental Protection Institute, Ministry of Agriculture and Rural Affairs, No. 31 Fukang Road, Nankai District, Tianjin 300191, China; ljyliangjiayin@163.com (J.L.); zhengxiangqun@126.com (X.Z.); 15620200536@163.com (T.N.); wang_jiar@163.com (J.W.); shenfeng@caas.cn (F.S.); 2 Key Laboratory of Rural Toilet and Sewage Treatment Technology, Ministry of Agriculture and Rural Affairs, No. 31 Fukang Road, Nankai District, Tianjin 300191, China; 3 Institute of Environment and Sustainable Development in Agriculture, No.12 Zhongguancun South Street, Haidian District, Beijing 100081, China

**Keywords:** propidium monoazide (PMA), real-time quantitative polymerase chain reaction (qPCR), metagenomic, biofilm viability, viable microbial community structure

## Abstract

Microbial community composition, function, and viability are important for biofilm-based sewage treatment technologies. Most studies of microbial communities mainly rely on the total deoxyribonucleic acid (DNA) extracted from the biofilm. However, nucleotide materials released from dead microorganisms may interfere with the analysis of viable microorganisms and their metabolic potential. In this study, we developed a protocol to assess viability as well as viable community composition and function in biofilm in a sewage treatment system using propidium monoazide (PMA) coupled with real-time quantitative polymerase chain reaction (qPCR) and metagenomic technology. The optimal removal of PMA from non-viable cells was achieved by a PMA concentration of 4 μM, incubation in darkness for 5 min, and exposure for 5 min. Simultaneously, the detection limit can reach a viable bacteria proportion of 1%, within the detection concentration range of 10^2^–10^8^ CFU/mL (colony forming unit/mL), showing its effectiveness in removing interference from dead cells. Under the optimal conditions, the result of PMA–metagenomic sequencing revealed that 6.72% to 8.18% of non-viable microorganisms were influenced and the composition and relative abundance of the dominant genera were changed. Overall, this study established a fast, sensitive, and highly specific biofilm viability detection method, which could provide technical support for accurately deciphering the structural composition and function of viable microbial communities in sewage treatment biofilms.

## 1. Introduction

The biological method is the predominant sewage treatment technology that removes biodegradable organic matter, nitrogen, phosphorus, and other pollutants by microorganisms [1,2]. The biofilm-based biological method is one of the primary technologies for biological sewage treatment at present [3,4]. The growth and development of biofilms in sewage treatment facilities can be generally divided into the following stages: the attachment of microorganisms on the carriers’ surfaces, production of extracellular polymeric substances (EPS), biofilm formation and maturity, and biofilm aging and detachment [5,6,7,8]. The community composition, function, and viability of biofilm are important to the removing efficiency of the treatment facilities [9,10,11,12,13,14]. In recent years, the development of sequencing technologies has extensively promoted knowledge of biofilm microbial community structure and function [15]. However, nucleotide materials released from dead microorganisms may interfere with the analysis of viable microorganisms and their metabolic potential especially when using DNA based methods such as PCR and sequencing [16]. Therefore, accessing the proportion of viable microorganisms and exploring their community composition and function in biofilms is crucial in studying the sewage treatment process [17,18].

Ethidium monoazide bromide (EMA), propidium iodide (PI), and propidium monoazide (PMA) are common nucleic acid stains frequently used for separating and identifying viable microorganisms [19]. Among these, PI is commonly used to assess the ratio of viable cells to dead or damaged ones through fluorescence microscopy, flow cytometry, and confocal laser scanning microscopy [20,21,22]. PMA and EMA can penetrate the cell membranes of dead or injured bacteria under intense light exposure, binding covalently to DNA, thereby hindering their PCR amplification reaction [23,24,25]. Since the integrity of cellular membranes is essential for organisms to sustain cellular metabolism and support life, the membrane-damage is commonly associated with the death of the bacteria. Therefore, the combination of PMA or EMA treatment with real-time PCR (qPCR) protocols can quantitatively detect viable microorganisms. Some prior studies have proposed that EMA may permeate the cell membranes of some viable cells, potentially resulting in an underestimation of both the quantity and composition of viable cells [24,25,26]. Hence, PMA coupled with qPCR (PMA-qPCR) emerges as more suitable for evaluating the composition and abundance of viable microorganisms within complex microbial systems.

PMA has been widely used in assessing the cellular viability of plant pathogens, foodborne pathogens, or pathogens from clinical settings [27,28,29,30]. For instance, Elizaquível et al. successfully revealed that viable and dead *Escherichia coli* (*E. coli*) O157:H7 can be successfully distinguished by the PMA-qPCR approach [31]. Jones et al. used a PMA-qPCR approach to detect and quantify viable *E. coli* from whole blood, and determined the lower limit of detection (LOD) was 10^2^ CFU/mL and linear range of quantification was 10^3^–10^8^ CFU/mL (R^2^ = 0.998) [32]. Reichelt et al. found that PMA-qPCR analysis can efficiently detect the viable but non-culturable (VBNC) state of *Campylobacters* in the environment of broiler farms [33].

In recent years, some researchers have treated sewage or sludge samples with PMA to reflect the structure and function of live bacteria more accurately [34,35]. However, the concentrations of PMA and the exposure times have varied a lot between different studies because different matrices in diverse sample types markedly affect the effectiveness of PMA treatment. For instance, Li et al. [36] treated sewage and sludge using a PMA concentration of 100 μM and a light exposure time of 4 min, and the results showed that matrices in sludge samples markedly reduced the effectiveness of PMA treatment. Liu et al. [37] treated the activated sludge mixed liquor suspended solids sample using a lower concentration of PMA (50 μM) and a longer blue light exposure (10 min) and found this condition could effectively shield 99.7–99.8% of the dead bacteria. Deshpande et al. [38] revealed that the observed microbial community structure and dominant flora of a rock and leaf biofilm were changed with 50 μM PMA treatment, and the treated samples had lower alpha diversity than the total DNA samples. Ni et al. [39] found that 5–30% of the genes in sludge samples were derived from non-viable or dead microorganisms, and these genes can be shielded after being treated with 0.1mM PMA and exposed to blue light for 15 min. The numbers of operational taxonomic units and Chao1 and Shannon indices of the sludge samples were significantly reduced with PMA treatment. A higher PMA treatment concentration would increase the cost and a shorter exposure time could impact the shield efficiency of dead cells. However, the application and optimization of PMA in sewage treatment biofilm is still lacking.

In this study, we developed a protocol to assess the viability of biofilm in a sewage treatment system, adding *E. coli* as the indicator strain (*E. coli* is one of the common indicator strains) [36,37,40,41,42]. PMA nucleic acid dye was used to shield dead microorganisms, and we optimized the optimal PMA concentration and exposure time to improve detection accuracy and reduce economic costs. In addition, we used the optimized PMA treatment protocol combined with qPCR to detect the viability of biofilm samples from a multi-layer composite media biofilter. Further, we revealed the viable community structure and function of the biofilms using PMA–metagenomic sequencing. This study established a fast, sensitive, and highly specific biofilm viability detection method, which could provide technical support for accurately deciphering the structural composition of viable microbial communities and their function in sewage treatment biofilms.

## 2. Materials and Methods

### 2.1. Bacterial Strains, Cultivation Conditions, and Preparation of Viable and Dead Cells

#### 2.1.1. Bacterial Strains and Cultivation Conditions

To determine the shielding efficiency of PMA on dead bacteria and the accuracy of qPCR results, *E. coli* was added as an indicator strain and the reasons are elaborated in Text S1. The *E. coli* HB101 (with streptomycin resistance, acquired from Shanghai Xin Yu Biotechnology Co., Ltd., Shanghai, China) was cultured in Luria–Bertani (LB) medium [43] and 30 mg/L streptomycin sulfate solution was added to avoid contamination. After 8 h incubation at 37 °C, the culture was spun at 150 rpm in a constant-temperature shaking incubator (Shanghai Yuejin Medical Equipment Co., Ltd., Shanghai, China) and a bacterial suspension was obtained. The suspension of *E. coli* HB101 was gradient diluted with 0.1 M phosphate buffered saline (PBS). Bacterial concentrations were quantified by spreading 100 µL of diluted samples on LB agar plates for 24 h at 37 °C and counting the CFU. The optical density at 600 nm (OD_600 nm_) was measured by an ultramicro nucleic acid detector (Implen GmbH Co., Ltd., Munich, Germany), and each gradient concentration of the *E. coli* suspension was used to establish the linear relationship between the determined OD_600 nm_ value and the concentration of the bacterial suspension. The results are shown in Text S2.

#### 2.1.2. Preparation of Viable and Dead Cells

The bacterial suspensions were adjusted to cell concentrations between 10^2^ and 10^8^ CFU/mL by serial dilutions. The suspension was divided into two aliquots. One aliquot was used as the viable sample (control group), and the other aliquot was used to prepare dead samples (inactivated group). To obtain dead cells, the suspension was heated in a water bath at 95 °C for 10 min [44,45,46]. To ensure the death of cells, 100 µL of heated *E. coli* HB101 was plated on NB plates with the same volume of *E. coli* HB101 without heat treatment on another plate as a positive control. The plates were placed at 37 °C and no colony growth was found after 24 h. 

### 2.2. Biological Filter Operation and Biofilm Sampling

#### 2.2.1. Biological Filter Operation

A multi-layer composite media biological filter was constructed to treat domestic sewage and the picture is shown in Figure S1. The size of the biological filter is 30 × 30 × 120 cm and the height of each layer of filter is 20 cm. The packing materials are zeolite (diameter 4–8 mm) and suspension spheres (diameter 10 cm) containing polyurethane sponges. The water outlet is 20 cm above the ground. We washed the packing materials several times before the experiment was started. We put the filler into the activated sludge to hang the film, and the biofilm was fixed to the filler and then filled into the biological filter (activated sludge was taken from the sewage treatment station of Baitansi Village, Tianjin, with a concentration of 2500 mg/L). The synthetic wastewater was composed of ammonium sulfate (NH_4_SO_4_), potassium dihydrogen phosphate (KH_2_PO_4_), and glucose (C_6_H_12_O_6_). The C/N/P of the inlet water was 100/5/1, the hydraulic load was 0.6 m^3^/m^2^/d, and the inlet water mode was continuous. Wind can flow through the ventilation pipe from the top to the bottom of the device, so that the upper part of the filter is in an aerobic state, and the lower part of the filter is immersed in water, which is in an anaerobic state. The biological filter was operated continuously for 120 days at room temperature (10–17 °C).

#### 2.2.2. Biofilm Sampling

The sewage treatment equipment continued to operate steadily until day 100 for biofilm sampling. Biofilm samples were taken at 5 cm, 10 cm, 30 cm, 50 cm, 70 cm, 90 cm, and 110 cm (from top to bottom) within the biological filter. Firstly, three parts of 5g biofilm packing materials were taken from different depths and shaken on 0.1 M PBS to release the biomass. Next, the packing materials were scraped with tweezers to obtain the remaining firmly adhered biofilm. Three parts of the biofilm suspension were mixed. We adjusted the concentration of the biofilm suspension with 0.1 M PBS to the OD_600 nm_ value measured in Section 2.1. To obtain dead cells, the biofilm suspension was heated in a water bath at 100 °C for 20 min.

### 2.3. PMA Condition Optimization

We took 500 μL of viable *E. coli* suspension and dead *E. coli* suspension (the concentration was 1 × 10^8^ CFU/mL [47]) and put them into 1.5mL transparent PVC centrifuge tubes, respectively, and then added 500 μL biofilm suspension. The mixtures were thoroughly vortexed. Different volumes of PMA solution (Upland Co., LTD.) were added to achieve final PMA concentrations of 0, 4, 8, 12, 16, and 20 μM. After thorough mixing, the samples were incubated in the dark at room temperature for 5 min and then exposed to light for 10 min using the LUYOR-3419 LED photoreactor (LUYOR INSTRUMENT CO, LTD). The samples were mixed once for 5 min. The LUYOR-3419 LED photoreactor provided uniform and maximal illumination to 24 × 1.5^−2^ mL vials, using illumination long-lasting LED lights with 465–475 nm emission from both sides and the bottom to all vials for efficient activation of PMA. The distance between the three light sources and vials was 5 mm, with a strength of 7900 μw/cm^2^ for each vacancy. Subsequently, they were centrifuged at 12,000 rpm for 2 min at 4 °C, and the supernatant was discarded while the sediment was retained for DNA extraction and qPCR detection. Three repetitions were performed in each condition.

Four-time gradients were selected for exposure times of 0, 5, 15, and 20 min, and the optimized PMA concentration was adopted. The other steps were the same as those for PMA concentration optimization. The three repetitions were performed in each condition.

### 2.4. PMA-qPCR Detection of Different Ratios of Viable/Dead Bacteria

Suspensions of viable and dead bacteria with a concentration of 1 × 10^8^ CFU/mL were taken and mixed in different ratios of viable to dead bacteria. In the 500 μL *E. coli* suspension, the proportions of viable bacteria were 0%, 1%, 10%, 20%, 40%, 60%, 80%, and 100%, respectively. The gradient solutions were treated with PMA under the optimized conditions outlined in Section 2.3. The samples were centrifuged at a speed of 12,000 rpm for 2 min at 4 °C. The supernatant was then discarded, leaving the precipitate for DNA extraction and qPCR detection.

### 2.5. PMA-qPCR Standard Curve

The cultured *E. coli* suspension was diluted with PBS solution to adjust the OD_600 nm_ of the cultured *E. coli* suspension, maintaining a bacterial concentration of 1 × 10^8^ CFU/mL. The bacterial suspension was gradient diluted with PBS solution to achieve concentrations of 1 × 10^8^, 1 × 10^7^, 1 × 10^6^, 1 × 10^5^, 1 × 10^4^, 1 × 10^3^, 1 × 10^2^, and 1 × 10^1^ CFU/mL, creating eight concentration gradients. A total of 500 μL of each bacterial suspension was taken and added to 1.5 mL transparent PVC centrifuge tubes, to which 500 μL of biofilm suspension with pre-determined OD values was added. The gradient solutions were treated with PMA under the optimized conditions outlined in Section 2.3. The samples were centrifuged at a speed of 12,000 rpm for 2 min at 4 °C. The supernatant was then discarded, leaving the precipitate for DNA extraction and qPCR detection. A standard curve of cycle threshold (Ct) values against the concentration of *E. coli* suspension (log10 CFU/mL) was plotted.

### 2.6. DNA Extraction

After PMA treatment, the suspension was centrifuged at a speed of 12,000 rpm for 2 min at a temperature of 4 °C. The supernatant was discarded, and DNA extraction from the precipitate followed the steps of the Qiagen DNeasy^®^ Powersoil^®^ Pro Kit (Qiagen, Frankfurt, Germany) (“MoBio PowerSoil kit” before acquisition from Qiagen) [48]. The extracted DNA was stored at −80 °C before detection.

### 2.7. Primer Specificity Verification and Establishment of qPCR Detection System

#### 2.7.1. Primer Specificity Verification

The primer sequences were as follows: size and specificity were verified using 1% agarose gel electrophoresis after PCR amplification with the provided *E. coli* DNA as a template, using the ABI SimpliAmp PCR instrument (Life Technologies Co., Ltd., Massachusetts, USA). The primer is based on the conserved gene ybiW of *E. coli*, referring to the local standard of Liaoning Province, DB21/T 2734.1-2017 “Method of PCR typing diagnosis for bacterial Part1: Method of PCR detection for *E. coli*” [49]. The result was shown in Text S3 and Figure S2. The primer sequences and PCR conditions are as follows:

Forward (F): 5’-GCGGCGTTGGAGAGTGATA-3’,
Reverse (R): 5’-AGCAATGGAAAAAGCAGGATG-3’.

The total volume of the PCR reaction system was 20 μL, with 2 μL of DNA template, 2.5 μL of PCR Buffer, 0.5 μL each of the upstream and downstream primers for *E. coli*, 2 μL of dNTP, 0.125 μL of Ex Taq DNA polymerase, and 17.375 μL of ddH_2_O. The PCR reaction consisted of 35 cycles: initial denaturation at 94 °C for 5 min, denaturation at 94 °C for 45 s, annealing at 56 °C for 45 s, extension at 72 °C for 50 s, and a final extension at 72 °C for 10 min.

#### 2.7.2. Establishment of qPCR Detection System

qPCR analysis used CFX96 Touch™ real-time PCR equipment (Bio-Rad, California, USA). The total volume of the qPCR reaction system was 20 μL, with 1 μL of DNA template, 10 μL of qPCR Mix, 0.4 μL each of the upstream and downstream primers for *E. coli*, and 8.2 μL of ddH_2_O. The qPCR conditions were as follows: a pre-denaturation step at 95 °C for 5 min, denaturation at 95 °C for 5 s, annealing at 55 °C for 20 s, and extension at 72 °C for 45 s, with a 40-cycle set, simultaneously collecting fluorescence signals. Melting curve analysis was performed from 65 to 95 °C, with a temperature increase of 0.5 °C every 5 s. Triplicates were set for each qPCR sample.

### 2.8. PMA Treatment for Viable Biofilm Quantification

Samples were taken at 5 cm, 10 cm, 30 cm, 50 cm, 70 cm, 90 cm, and 110 cm (from top to bottom) within the biofilter, denoted as D5, D10, D30, D50, D70, D90, and D110, respectively. We adjusted the concentration of the sample to 1 × 10^8^ CFU/mL. The biofilm bacterial suspensions obtained from each depth were treated with PMA under the optimized conditions outlined in Section 2.3., denoted as D5-PMA, D10-PMA, D30-PMA, D50-PMA, D70-PMA, D90-PMA, and D110-PMA, respectively. The samples were centrifuged at a speed of 12,000 rpm for 2 min at 4 °C. The supernatant was then discarded, leaving the precipitate for DNA extraction and qPCR detection. The bacterial concentrations were calculated using the standard curve synthesized in Section 2.5. The formula for calculating the proportion of viable bacteria was as follows:

Proportion of viable bacteria = (34.159 − Ct_1_)/(34.159 − Ct_2_) × 100% (1)

Ct_1_: Ct values of biofilm samples with PMA treatment.

Ct_2_: Ct values of biofilm samples without PMA treatment.

34.159: According to the regression equation.

### 2.9. Metagenomic Sequencing and Analysis

Metagenomic sequencing was performed on six biofilm samples, namely D5, D10, D110, D5-PMA, D10-PMA, and D110-PMA. DNA extracts were fragmented to an average size of about 400 bp using Covaris M220 (Gene Company Limited, Beijing China) for paired-end library construction. Paired-end sequencing was performed on Illumina Novaseq 6000 (Illumina Inc., San Diego, CA, USA) at Majorbio Bio-Pharm Technology Co., Ltd. (Shanghai, China). We acquired 6G sequence data for each sample. The data were analyzed on the free online platform of Majorbio Cloud Platform (www.majorbio.com). The paired-end Illumina reads were trimmed of adaptors, and low-quality reads (length < 50 bp or with a quality value < 20 or having N bases) were removed by fastp [50] (https://github.com/OpenGene/fastp, version 0.20.0, 4 December 2020). The metagenomics data were assembled using MEGAHIT [51] (https://github.com/voutcn/megahit, version 1.1.2, 1 August 2017). Contigs with a length ≥ 300 bp were selected as the final assembling result, and then the contigs were used for further gene prediction and annotation.

Open reading frames (ORFs) from each assembled contig were predicted using Prodigal [52]/MetaGene [53] (http://metagene.cb.k.u-tokyo.ac.jp/, version 2.6.3, February 17, 2016). The predicted ORFs with a length ≥ 100 bp were retrieved and translated into amino acid seq ences using the NCBI translation table (http://www.ncbi.nlm.nih.gov/Taxonomy/taxonomyhome.html/index.cgi?chapter=tgencodes#SG1, 10 August 2023). A non-redundant gene catalog was constructed using CD-HIT [54] (http://www.bioinformatics.org/cd-hit/, version 4.6.1, 1 September 2009) with 90% sequence identity and 90% coverage. High-quality reads were aligned to the non-redundant gene catalogs to calculate gene abundance with 95% identity using SOAPaligner [55] (http://soap.genomics.org.cn/, version 2.21, 23 December 2013).

Representative sequences of non-redundant gene catalogs were aligned to the NR database with an e-value cutoff of 1 × 10^−5^ using Diamond [56] (http://www.diamondsearch.org/index.php, version 0.8.35, 4 February 2017) for taxonomic annotations. The KEGG annotation was conducted using Diamond [56] (http://www.diamondsearch.org/index.php, version 0.8.35, 4 February 2017) against the Kyoto Encyclopedia of Genes and Genomes database (http://www.genome.jp/keeg/, 1 August 2023) with an e-value cutoff of 1 × 10^−5^.

### 2.10. Statistical Analysis

Bar and line charts were generated using Origin 2023. The indicated significant differences (*p* < 0.05) among heat-inactivated *E. coli* treatments and viable *E. coli* treatments were determined by analysis of variance (ANOVA), using SPSS 25.0. Species analysis was conducted at the genus level using the free online platform of Majorbio Cloud Platform (www.majorbio.com). We conducted a significant difference analysis of microbial composition and function between PMA-treated samples and untreated samples (*p* < 0.05), using the Fisher exact test.

## 3. Results

### 3.1. Optimization of PMA Conditions

#### 3.1.1. Optimization of PMA Concentration

**Figure 1:**
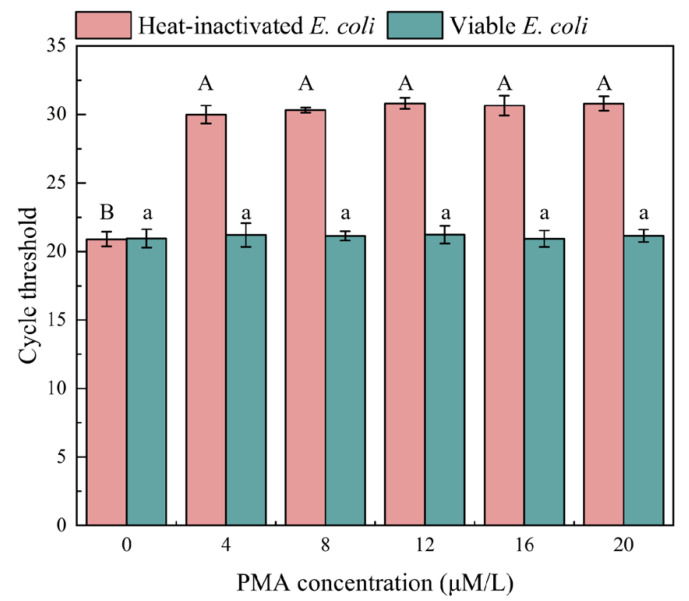
The effect of PMA concentration on qPCR amplification results. Note: different capital letters indicate significant differences among heat-inactivated *E. coli* treatments (*p* < 0.05), different lowercase letters indicate significant differences among viable *E. coli* treatments (*p* < 0.05).

**Figure 2:**
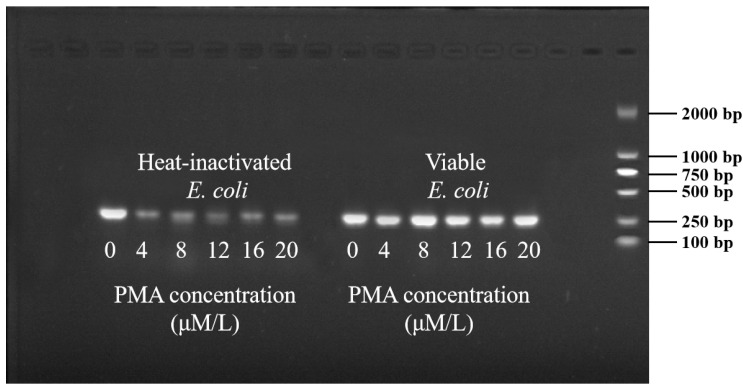
Gel electrophoresis diagram of PMA concentration on qPCR amplification results.

Different concentrations of PMA were applied to the suspension of biofilms marked with heat-inactivated and viable *E. coli*, and their impact on qPCR amplification was investigated. As shown in Figure 1, PMA treatment significantly inhibited the DNA amplification of heat-inactivated *E. coli*. The Ct value of the untreated sample exhibited a notable difference compared to PMA-treated samples. When the PMA concentration was 4 μM, the Ct value reached 30 and was obviously higher than the untreated one (PMA = 0 μM, Ct = 20.9). The Ct value could not further increase when the PMA concentration exceeded 4 μM. Also, the change in PMA concentration did not inhibit the qPCR amplification of live *E. coli* (*p* < 0.05). Therefore, at the PMA concentration of 4 μM, it could completely bind to the DNA of heat-inactivated *E. coli*, thereby inhibiting DNA amplification. Considering the dosage and economic factors of PMA, 4 μM was chosen as the optimal PMA concentration for rapid detection of biofilm viability. As shown in Figure 2, when the PMA concentration exceeded 4 μM, the bands of the agarose gel of dead bacteria were darkened, with no effect on the bands of the agarose gel of viable bacteria.

#### 3.1.2. Optimization of Exposure Time

**Figure 3:**
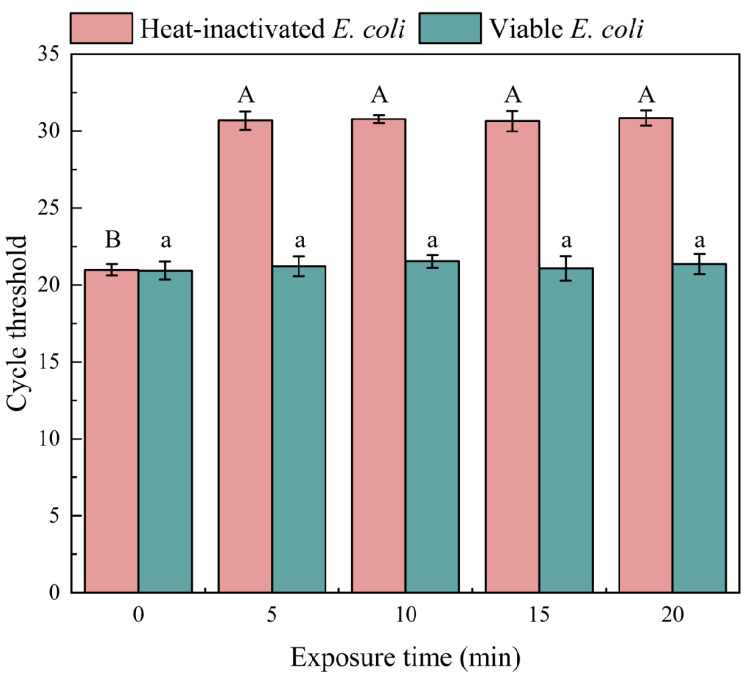
The effect of exposure time on qPCR amplification results. Note: different capital letters indicate significant differences among heat-inactivated *E. coli* treatments (*p* < 0.05), different lowercase letters indicate significant differences among viable *E. coli* treatments (*p* < 0.05).

Based on the optimal PMA concentration determined in Section 3.1.1, 4 μM of PMA was added, and we then investigated the impact of different blue light exposure times on qPCR amplification results. As shown in Figure 3, exposure to blue light for 5 min significantly inhibited the DNA amplification of heat-inactivated *E. coli*. (Ct = 20.99). When the exposure time exceeded 5 min, there was no significant change in Ct values (*p* < 0.05). Within the 0–20 min exposure range, there was no significant variation in the Ct values of viable *E. coli* qPCR amplification (*p* < 0.05), indicating that different exposure times did not affect the DNA amplification of viable *E. coli*. Therefore, when the exposure time was 5 min, PMA could completely bind to the DNA of heat-inactivated *E. coli*, thereby inhibiting DNA amplification. Considering experimental time and economic factors, 5 min of exposure was chosen as the optimal exposure time for rapid detection of biofilm viability.

### 3.2. Selective Amplification of Living Cells by PMA-qPCR With Different Viable/Dead Bacterial Ratio Mixtures

**Figure 4:**
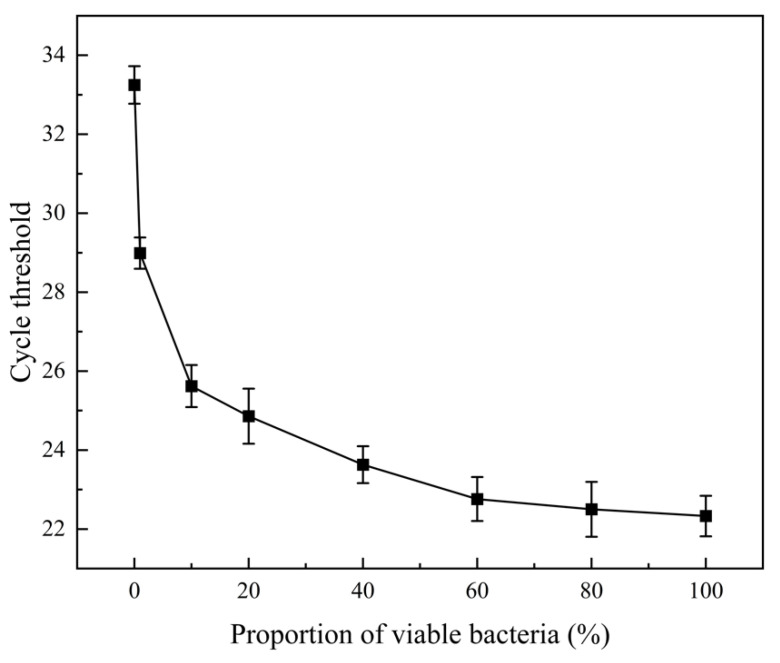
The impact of different proportions of viable bacteria on qPCR amplification results.

Suspensions of dead and viable bacteria at a concentration of 1 × 10^8^ CFU/mL were mixed in different viable/dead ratios. As the proportion of viable bacteria increased, a gradual decrease in Ct values was observed (Figure 4). The detection limit can be reached when the proportion of viable bacteria is 1%. When the concentration of viable bacteria was 0%, the cycle number (33.4) was an incredible signal, and it is not possible to distinguish whether it is generated by viable cells. For each experiment, the matrix in the biofilm samples from different batches may have affected the efficiency of DNA extraction and qPCR amplification. Therefore, PMA-qPCR could effectively distinguish between viable and dead cells in biofilms labeled with *E. coli* as the marker strain. In a mixed system of viable and dead bacteria, PMA selectively inhibited the DNA of dead cells, enabling effective amplification of DNA from viable cells. Traditional qPCRs amplify all DNA in mixed systems containing viable and dead cells, leading to overestimation of the diversity and composition of viable microorganisms in the system. Therefore, it was demonstrated that PMA-qPCR could rapidly quantify the viable cells in biofilm.

### 3.3. Evaluation of PMA-qPCR Standard Curve and Detection Limit

**Figure 5:**
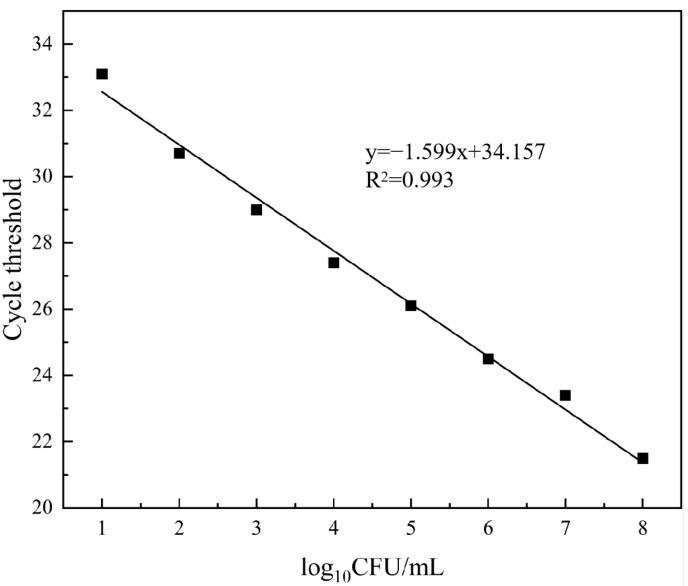
PMA-qPCR standard curve.

A suspension of *E. coli* with a concentration of 1 × 10^8^ CFU/mL was subjected to eight consecutive dilutions to obtain a standard curve. The correlation between the concentration of *E. coli* suspension and Ct values is depicted in Figure 5. There was a linear correlation between the concentration of *E. coli* suspension and Ct values, represented by the equation y = −1.599x + 34.157, R^2^ = 0.993 with a detection range concentration of 10^2^–10^8^ CFU/mL. This detection range is consistent with that reported by Fu et al. [57] for *E. coli* O157:H7 in pure culture systems.

### 3.4. Detection of the Viability of Biofilm Samples

**Figure 6:**
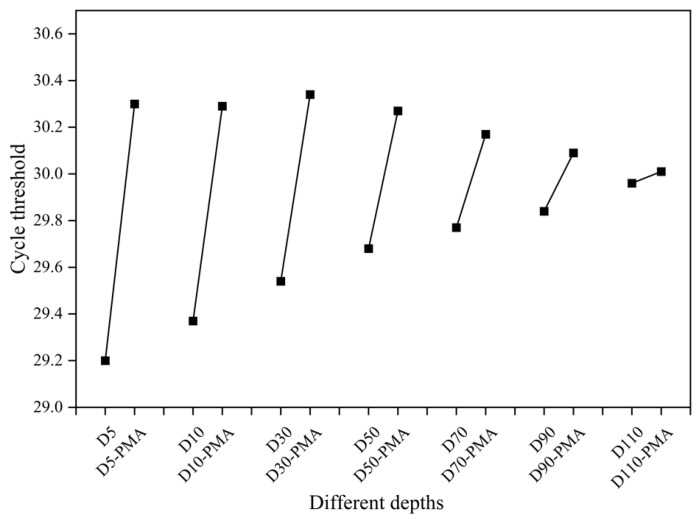
qPCR detection results of sewage treatment biofilm samples without and with PMA treatment.

The viability of seven biofilm samples from a multi-layer composite media biofilter was detected using the optimized PMA treatment protocol combined with qPCR. And the results are shown in Figure 6. The Ct values of the biofilm bacterial suspensions with PMA treatment were higher than those of the untreated biofilm bacterial suspensions, indicating that PMA treatment effectively masked the presence of dead bacteria in the biofilm.

**Figure 7:**
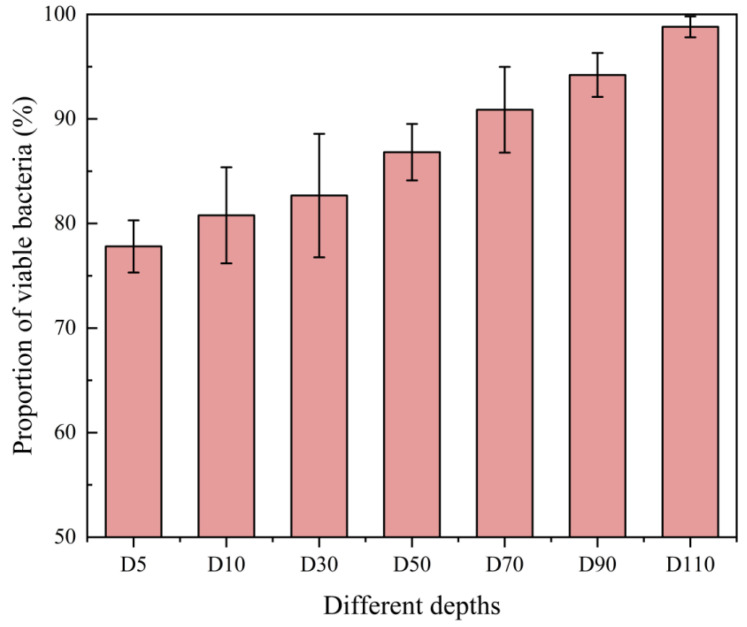
The proportion of viable bacteria in biofilm samples.

The optimized PMA treatment protocol combined with qPCR could effectively reflect the proportion of viable bacteria in biofilms. As shown in Figure 7, the proportions of viable bacteria at different depths were different. The proportion of viable bacteria in the biofilm samples increased with increasing depth and ranged from 78% to 98%. The viability of the biofilm increased with increasing depth. Since the flow direction of the biofilter is top–down (as shown in Figure S1), the pollution load shows a trend of decreasing gradually with the increase in depth. The high load of pollutants in the upper layer leads to faster metabolism of the biofilm. At the same time, the ventilation volume in the upper layer is larger, and the influence of wind shear leads to more dead cells in the upper layer.

### 3.5. Microbial Community Structure and Function of Sewage Treatment Biofilm without and with PMA Treatment

#### 3.5.1. Microbial Community Structure of Sewage Treatment Biofilm without and with PMA Treatment

**Figure 8:**
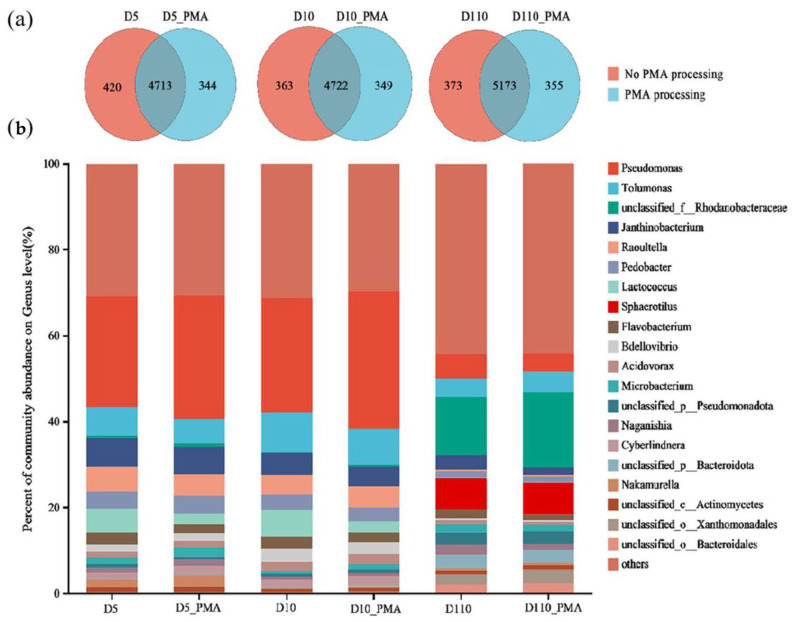
The genus-level microbial community structure of sewage treatment biofilm without and with PMA treatment. (**a**) Venn diagram of microbial genera of PMA-treated and -untreated samples. (**b**) The genus-level microbial community composition without and with PMA treatment.

Changes in the number of unique genera in the biofilm microbiota occurred without and with PMA treatment (Figure 8a), indicating that, with PMA treatment, non-viable microorganisms were shielded and viable microorganisms were amplified, leading to significant changes in the microbial community structure. The total number of genera decreased by 1.48%, 0.28%, and 0.32% with PMA treatment. Without PMA treatment, the number of unique genera of biofilm microbiota at depths of 5 cm, 10 cm, and 110 cm on the filler surface was 420, 363, and 373, respectively, accounting for 8.18%, 7.18%, and 6.72% of the total number of microbial genera. With PMA treatment, the number of unique genera of biofilm microbiota at depths of 5 cm, 10 cm, and 110 cm on the filler surface was 344, 349, and 355, respectively, accounting for 6.80%, 6.88%, and 6.42% of the total number of microbial genera.

The relative abundance of the top 20 genera in the microbial community composition at depths of 5 cm, 10 cm, and 110 cm on the surface of the material without and with PMA treatment of the biofilm is shown in Figure 8b. There were changes in the composition and relative abundance of dominant genera at the genus level without and with PMA treatment of the biofilm. For instance, at a depth of 5 cm, the abundance of the top five dominant genera of the biofilm were *Pseudomonas* (25.78%), *Tolumonas* (6.71%), *Janthinobacterium* (6.71%), *Raoultella* (5.73%), and *Lactococcus* (5.52%) without PMA treatment. With PMA treatment, the relative abundance of *Pseudomonas* was increased by 2.9% and the relative abundance of *Tolumonas*, *Janthinobacterium*, *Raoultella*, and *Lactococcus* was decreased by 1.01%, 0.40%, 0.64%, and 2.91% respectively. At a depth of 10 cm, after treatment by PMA, the relative abundance of *Pseudomonas* and *Raoultella* increased by 5.47% and 0.57%, respectively, while that of *Tolumonas*, *Lactococcus*, and *Janthinobacterium* decreased by 0.87%, 0.55%, and 3.69%, respectively. At a depth of 110 cm, without PMA treatment, the top five dominant genera were *unclassified_f_Rhodanobacteraceae* (13.63%), *Sphaerotilus* (7.38%), *Pseudomonas* (5.60%), *Tolumonas* (4.38%), and *Janthinobacterium* (3.26%). With PMA treatment, the relative abundance of *unclassified_f_Rhodanobacteraceae* and *Tolumonas* increased by 3.85% and 0.38%, respectively, while that of *Sphaerotilus*, *Pseudomonas*, and *Janthinobacterium* decreased by 0.04%, 1.25%, and 1.49%, respectively. The abundance of *unclassified_o_Xanthomonadales* (3.11%) exceeded *Janthinobacterium* with PMA treatment.

**Figure 9:**
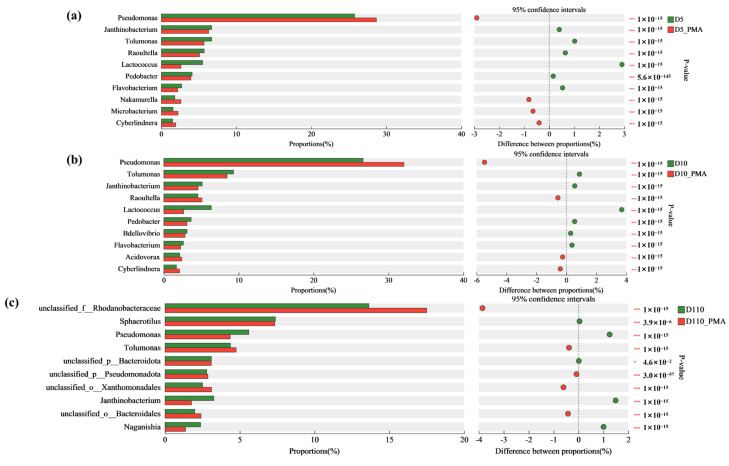
Differences in the composition of microbial community structure at the genus level of sewage treatment biofilm without and with PMA treatment. (**a**) The depth is 5 cm. (**b**) The depth is 10 cm. (**c**) The depth is 110 cm. 0.01 < *p* ≤ 0.05 *, *p* ≤ 0.001 ***.

**Figure 10:**
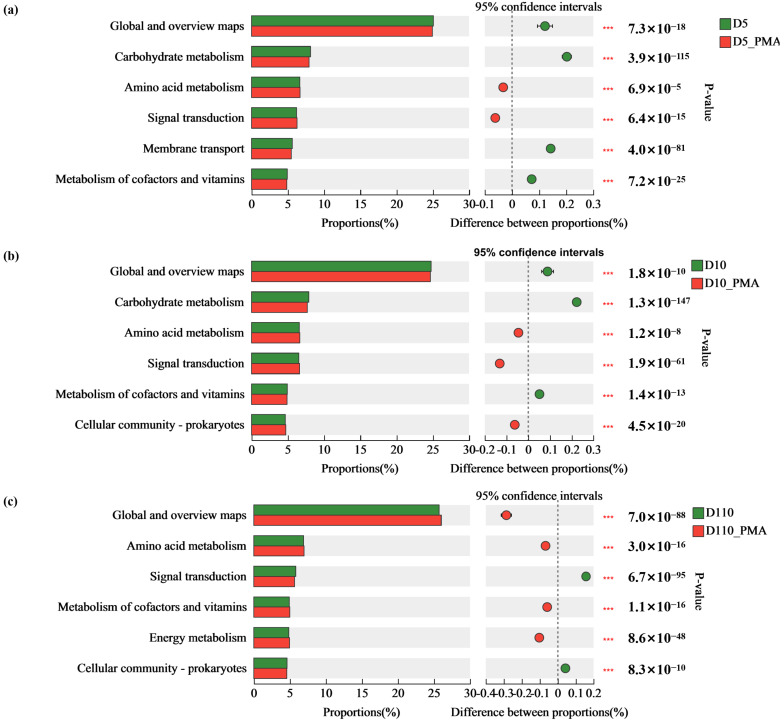
Differences in microbial functions of sewage treatment biofilm between without and with PMA treatment (KEGG functional, level 2). (**a**) The depth is 5 cm. (**b**) The depth is 10 cm. (**c**) The depth is 110 cm. *p* ≤ 0.001 ***.

We used the Fisher exact test to further distinguish the different genera with and without treatment. Interestingly, similar changes were observed in the biofilm at depths of 5cm and 10 cm which were different from that in D110. For examples, with PMA treatment, the relative abundance of *Pseudomonas* and *Cyberlindnera* increased, while the relative abundance of *Tolumonas*, *Janthinobacteriurn*, *Lactococcus*, *Pedobacter,* and *Flavobacterium* decreased (Figure 9). In the biofilm at depths of 110 cm, nine of the top ten genera had significant differences with and without treatment (Figure 10) and the relative abundance of *Naganishia*, *Sphaerotilus,* and *Pscudomonasd* decreased, while that of *Tolumonas* increased. These results indicated that the death in these biofilms does not happen in unbiased ways, and the live/death community was different in D5 and D10 (with higher pollution load and more oxygen) and in D110 (with low pollution load and less oxygen).

#### 3.5.2. Microbial Community Function of Sewage Treatment Biofilm without and with PMA Treatment

We used the Fisher exact test to further distinguish the different microbial functions of sewage treatment biofilm between samples with and without treatment. Consistent with the observation in the microbial community structure, we found the change in function with and without PMA treatment was also similar between D5 and D10. In biofilms at depths of 5 cm and 10 cm, the relative abundance of “Global and overview maps”, “Carbohydrate metabolism” and “Metabolism of cofactors and vitamins” decreased in the PMA-treated group compared with the PMA-untreated group, and the relative abundance of “Amino acid metabolism” and “Signal transduction” increased. However, the relative abundance of “Global and overview maps” and “Metabolism of cofactors and vitamins” in the 110 cm biofilm increased, while the relative abundance of “Signal transduction” decreased (Figure 10).

## 4. Discussion

Previous studies of microbial communities in sewage treatment biofilms have typically extracted total DNA directly from the biofilm. However, during biofilm formation, microorganisms undergo continuous processes of adsorption, growth, decay, and detachment [4,58,59]. This direct DNA extraction approach cannot differentiate viable from non-viable microorganisms, leading to an overestimation of their role in the biofilm. Microbial DNA consists of extracellular DNA (eDNA) and intracellular DNA (iDNA), and eDNA is predominantly found in non-viable cells [60,61,62]. Some researchers remove eDNA and extract iDNA to represent nucleic acids of viable cells. However, iDNA extraction procedures are complex and less commonly applied in research. Additionally, the presence of DNase inhibitors in environmental samples may interfere with the removal of eDNA [63,64,65]. Therefore, utilizing PMA-qPCR combined techniques for detecting viable microorganisms in biofilms is more convenient and offers higher sensitivity and specificity.

In this study, *E. coli* was used as the marker, the optimal concentration of PMA was determined to be 4 μM, and the optimal exposure time was determined to be 5 min by optimizing the PMA treatment conditions. Meanwhile, the results showed that selective expansion of viable cells could be achieved under the optimized best PMA treatment conditions. Fu et al. showed that the PMA concentration of 50 μM and the exposure time of 15 min were the optimal conditions for studying the formation and eradication of dormant *E. coli* O157:H7 in agricultural soils [57], in agreement with the findings of Elizaquivel et al. [31]. Ni et al. [66] treated sludge from anaerobic digestion tanks with 0.1 mM PMA, with an exposure time set at 15 min, and investigated the viable microbial composition. Furthermore, under the same PMA concentration and exposure time, changes in the viable microbial community in anaerobic membrane bioreactors treating low-temperature domestic sewage have been revealed using PMA-PCR technology [66]. Therefore, this study reduced the concentration and exposure time of PMA by optimizing the treatment conditions, shortened the extraction period of viable microbial DNA, and lowered experimental costs.

However, it is worth noting that the PCR fluorescence signal did not completely disappear with the increase in PMA concentration (Figure 2). Previous studies have shown that it is impossible to completely neutralize nucleic acids from dead microorganisms [67]. A total signal neutralization of dead cells remains one of the greatest challenges to be resolved for PMA-qPCR, because a multitude of factors limits the efficacy of the technique to exclude DNA from dead cells, including the cell concentration and properties, photolysis time, wavelength, presence of facilitating agents, qPCR primer properties, length of the DNA sequence amplified, and so on [68]. To better differentiate dead and viable cells, we defined Ct values higher than 32 as incredible signals. At the same time, when the concentration was 10 CFU/mL the corresponding Ct value was 33.2. Therefore, the detectable range of this experiment is 10^2^–10^8^ CFU/mL.

The presence of non-viable microorganisms affects the composition and metabolic functional analysis of microbial communities. PMA treatment altered the number of unique genera in the biofilm samples. The unique genera in PMA-untreated samples represent the dead genera. The unique genera in PMA-treated samples represent genera that were not detected without treatment. The undetectable nature of these genera maybe related to their low abundance in the samples. In the three samples, 6.72% to 8.18% of non-viable microorganisms were influenced by PMA, consistent with the comparative analysis of total DNA and viable DNA in anaerobic nitrification tanks by Vrieze et al. [17]. Successful shielding of non-viable microorganisms may result in their increased or decreased abundance [69,70]. In this study, without and with PMA treatment, the microbial community structures at the genus level of the biofilm were similar in both viable microbial communities and total microbial communities, but there were changes in the composition and relative abundance of the top five dominant genera. In a word, there were significant differences in the composition and function of biofilms. The method of using PMA to shield non-viable microorganisms will improve the accuracy of subsequent microbial composition and metabolic functional analysis [71].

## 5. Conclusions

The optimization of PMA treatment conditions has been the focus of many researchers, because the effectiveness of PMA treatment is markedly affected by the different matrices in different samples. In this study, we found the optimal PMA condition was 4 μM/L, incubation in darkness for 5 min, and exposure for 5 min. The detection limit can reach a viable bacteria proportion of 1%, and within the detection concentration range of 10^2^–10^8^ CFU/mL. The experiment time was much shorter, and the cost was reduced. In addition, a combination of PMA treatment using the optimal conditions and metagenomic methods was utilized to assess biofilm viability in sewage treatment, offering the advantages of accuracy, speed, efficiency, and robust specificity. We observed significant differences in microbial composition and function between viable community (PMA-treated) and total community (untreated groups). For example, the functional genes of “Global and overview maps”, “Carbohydrate metabolism”, and “Metabolism of cofactors and vitamins” in aerobic biofilm were decreased after PMA treatment, while the “Global and overview maps” and “Metabolism of cofactors and vitamins” were increased in anaerobic biofilm after PMA treatment. Overall, this work provides technical support for in-depth exploration of functional microorganisms.

## Data Availability

Data are contained within the article.

## Supplementary Materials

The following supporting information can be downloaded at: https://www.mdpi.com/article/10.3390/microorganisms12081508/s1, Text S1: The reason for using *E. coli* as indicator strain. Text S2: Determination of concentration of *E. coli* suspension [72]. Text S3: Specificity verification of primers. Figure S1: Multi-layer composite filler biological filter. Figure S2: Gel electrophoresis diagram of E. coli primer specificity verification.

## References

[1] Sanchez-Huerta C., Medina J.S., Wang C., Fortunato L., Hong P.-Y. (2023). Understanding the role of sorption and biodegradation in the removal of organic micropollutants by membrane aerated biofilm reactor (MABR) with different biofilm thickness. Water Res..

[2] Liang C., de Jonge N., Carvalho P.N., Nielsen J.L., Bester K. (2021). Biodegradation kinetics of organic micropollutants and microbial community dynamics in a moving bed biofilm reactor. Chem. Eng. J..

[3] Nelson M.J., Nakhla G., Zhu J. (2017). Fluidized-Bed Bioreactor Applications for Biological Wastewater Treatment: A Review of Research and Developments. Engineering.

[4] Derlon N., Coufort-Saudejaud C., Queinnec I., Paul E. (2013). Growth limiting conditions and denitrification govern extent and frequency of volume detachment of biofilms. Chem. Eng. J..

[5] Huang H., Ren H., Ding L., Geng J., Xu K., Zhang Y. (2014). Aging biofilm from a full-scale moving bed biofilm reactor: Characterization and enzymatic treatment study. Bioresour. Technol..

[6] Simões M., Simões L.C., Vieira M.J. (2010). A review of current and emergent biofilm control strategies. LWT—Food Sci. Technol..

[7] Zhao Y., Zhu S., Fan X., Zhang X., Ren H., Huang H. (2022). Precise portrayal of microscopic processes of wastewater biofilm formation: Taking SiO2 as the model carrier. Sci. Total Environ..

[8] Wang J., Liu Q., Wu B., Zhao F., Ma S., Hu H., Zhang X., Ren H. (2019). Quorum sensing signaling distribution during the development of full-scale municipal wastewater treatment biofilms. Sci. Total Environ..

[9] Reboleiro-Rivas P., Martín-Pascual J., Juárez-Jiménez B., Poyatos J.M., Vílchez-Vargas R., Vlaeminck S.E., Rodelas B., González-López J. (2015). Nitrogen removal in a moving bed membrane bioreactor for municipal sewage treatment: Community differentiation in attached biofilm and suspended biomass. Chem. Eng. J..

[10] Qiu Z., Zhang S., Ding Y., Zhang W., Gong L., Yuan Q., Mu X., Fu D. (2021). Comparison of Myriophyllum Spicatum and artificial plants on nutrients removal and microbial community in constructed wetlands receiving WWTPs effluents. Bioresour. Technol..

[11] Chen Y., Wang Y., Paez-Espino D., Polz M.F., Zhang T. (2021). Prokaryotic viruses impact functional microorganisms in nutrient removal and carbon cycle in wastewater treatment plants. Nat. Commun..

[12] Han F., Wei D., Ngo H.H., Guo W., Xu W., Du B., Wei Q. (2018). Performance, microbial community and fluorescent characteristic of microbial products in a solid-phase denitrification biofilm reactor for WWTP effluent treatment. J. Environ. Manag..

[13] Roveto P.M., Gupta A., Schuler A.J. (2021). Effects of surface skewness on local shear stresses, biofilm activity, and microbial communities for wastewater treatment. Bioresour. Technol..

[14] Campo R., Vassallo A., Rabbeni G., Arancio W., Gallo G., Di Bella G. (2021). Reactivation of aerobic granular sludge for the treatment of industrial shipboard slop wastewater: Effects of long-term storage on granules structure, biofilm activity and microbial community. J. Water Process Eng..

[15] Wensel C.R., Pluznick J.L., Salzberg S.L., Sears C.L. (2022). Next-generation sequencing: Insights to advance clinical investigations of the microbiome. J. Clin. Investig..

[16] Nagler M., Podmirseg S.M., Mayr M., Ascher-Jenull J., Insam H. (2021). The masking effect of extracellular DNA and robustness of intracellular DNA in anaerobic digester NGS studies: A discriminatory study of the total DNA pool. Mol. Ecol..

[17] De Vrieze J., Pinto A.J., Sloan W.T., Ijaz U.Z. (2018). The active microbial community more accurately reflects the anaerobic digestion process: 16S rRNA (gene) sequencing as a predictive tool. Microbiome.

[18] De Vrieze J., Raport L., Roume H., Vilchez-Vargas R., Jáuregui R., Pieper D.H., Boon N. (2016). The full-scale anaerobic digestion microbiome is represented by specific marker populations. Water Res..

[19] Yang B., Dai J., Zhao Y., Wu J., Ji C., Zhang Y. (2022). Advances in preparation, application in contaminant removal, and environmental risks of biochar-based catalysts: A review. Biochar.

[20] Stiefel P., Schmidt-Emrich S., Maniura-Weber K., Ren Q. (2015). Critical aspects of using bacterial cell viability assays with the fluorophores SYTO9 and propidium iodide. BMC Microbiol..

[21] Liu L., Liu C., Zhang H., He J., Zhai J., Yu D., Dong S. (2020). How to Identify the “LIVE/DEAD” States of Microbes Related to Biosensing. ACS Sens..

[22] Hua X.-W., Bao Y.-W., Wang H.-Y., Chen Z., Wu F.-G. (2017). Bacteria-derived fluorescent carbon dots for microbial live/dead differentiation. Nanoscale.

[23] Pan Y., Breidt F. (2007). Enumeration of viable *Listeria monocytogenes* cells by real-time PCR with propidium monoazide and ethidium monoazide in the presence of dead cells. Appl. Environ. Microbiol..

[24] Vondrakova L., Turonova H., Scholtz V., Pazlarova J., Demnerova K. (2018). Impact of various killing methods on EMA/PMA-qPCR efficacy. Food Control.

[25] Reyneke B., Ndlovu T., Khan S., Khan W. (2017). Comparison of EMA-, PMA- and DNase qPCR for the determination of microbial cell viability. Appl. Microbiol. Biotechnol..

[26] Nocker A., Cheung C.-Y., Camper A.K. (2006). Comparison of propidium monoazide with ethidium monoazide for differentiation of live vs. dead bacteria by selective removal of DNA from dead cells. J. Microbiol. Methods.

[27] Li F., Li F., Yang G., Aguilar Z.P., Lai W., Xu H. (2018). Asymmetric polymerase chain assay combined with propidium monoazide treatment and unmodified gold nanoparticles for colorimetric detection of viable emetic Bacillus cereus in milk. Sens. Actuators B Chem..

[28] Al-Daoud F., Gossen B.D., Robson J., McDonald M.R. (2017). Propidium Monoazide Improves Quantification of Resting Spores of *Plasmodiophora brassicae* with qPCR. Plant Dis..

[29] Hanifian S. (2020). Behavior of Mycobacterium avium paratuberculosis in Lighvan cheese tracked by propidium monoazide qPCR and culture. LWT.

[30] Tantikachornkiat M., Sakakibara S., Neuner M., Durall D.M. (2016). The use of propidium monoazide in conjunction with qPCR and Illumina sequencing to identify and quantify live yeasts and bacteria. Int. J. Food Microbiol..

[31] Elizaquivel P., Azizkhani M., Sanchez G., Aznar R. (2013). Evaluation of *Zataria multiflora Boiss.* essential oil activity against *Escherichia coli* O157:H7, *Salmonella enterica* and *Listeria monocytogenes* by propidium monoazide quantitative PCR in vegetables. Food Control.

[32] Jones K.L., Cunha F., Casaro S., Galvao K.N. (2024). Optimization and Testing of a Commercial Viability PCR Protocol to Detect *Escherichia coli* in Whole Blood. Microorganisms.

[33] Reichelt B., Szott V., Stingl K., Roesler U., Friese A. (2023). Detection of Viable but Non-Culturable (VBNC)-*Campylobacter* in the Environment of Broiler Farms: Innovative Insights Delivered by Propidium Monoazide (PMA)-v-qPCR Analysis. Microorganisms.

[34] Krohn C., Jansriphibul K., Dias D.A., Rees C.A., van den Akker B., Boer J.C., Plebanski M., Surapaneni A., O’Carroll D., Richard S. (2024). Dead in the water—Role of relic DNA and primer choice for targeted sequencing surveys of anaerobic sewage sludge intended for biological monitoring. Water Res..

[35] Eramo A., Morales Medina W.R., Fahrenfeld N.L. (2019). Viability-based quantification of antibiotic resistance genes and human fecal markers in wastewater effluent and receiving waters. Sci. Total Environ..

[36] Li D., Tong T., Zeng S., Lin Y., Wu S., He M. (2014). Quantification of viable bacteria in wastewater treatment plants by using propidium monoazide combined with quantitative PCR (PMA-qPCR). J. Environ. Sci..

[37] Liu W., Xiang P., Ji Y., Chen Z., Lei Z., Huang W., Huang W., Liu D. (2023). Response of viable bacteria to antibiotics in aerobic granular sludge: Resistance mechanisms and behaviors, bacterial communities, and driving factors. Water Res..

[38] Deshpande A.S., Fahrenfeld N.L. (2023). Influence of DNA from non-viable sources on the riverine water and biofilm microbiome, resistome, mobilome, and resistance gene host assignments. J. Hazard. Mater..

[39] Ni J., Hatori S., Wang Y., Li Y.-Y., Kubota K. (2019). Uncovering Viable Microbiome in Anaerobic Sludge Digesters by Propidium Monoazide (PMA)-PCR. Microb. Ecol..

[40] Zhang X., Lu W., Han E., Wang S., Shen J. (2014). Hybrid Nanostructure-based Immunosensing for Electrochemical Assay of Escherichia coli as Indicator Bacteria Relevant to the Recycling of Urban Sludge. Electrochim. Acta.

[41] Cooksey E.M., Singh G., Scott L.C., Aw T.G. (2019). Detection of coliphages and human adenoviruses in a subtropical estuarine lake. Science of The Total Environment.

[42] Kapoor V., Elk M., Toledo-Hernandez C., Santo Domingo J.W. (2017). Analysis of human mitochondrial DNA sequences from fecally polluted environmental waters as a tool to study population diversity. AIMS Environ. Sci..

[43] Nakanishi A., Omino N., Nakamura T., Goto S., Matsumoto R., Yomogita M., Narisawa N., Kimijima M., Iritani K. (2024). Evaluation of Cellular Responses of Heterotrophic *Escherichia coli* Cultured with Autotrophic *Chlamydomonas reinhardtii* as a Nutrient Source by Analyses Based on Microbiology and Transcriptome. Microorganisms.

[44] Liu Y., Schulze-Makuch D., de Vera J.-P., Cockell C., Leya T., Baque M., Walther-Antonio M. (2018). The Development of an Effective Bacterial Single-Cell Lysis Method Suitable for Whole Genome Amplification in Microfluidic Platforms. Micromachines.

[45] Karni M., Zidon D., Polak P., Zalevsky Z., Shefi O. (2013). Thermal Degradation of DNA. DNA Cell Biol..

[46] Macori G., McCarthy S.C., Burgess C.M., Fanning S., Duffy G. (2020). Investigation of the Causes of Shigatoxigenic *Escherichia coli* PCR Positive and Culture Negative Samples. Microorganisms..

[47] Guo L., Ze X., Feng H., Liu Y., Ge Y., Zhao X., Song C., Jiao Y., Liu J., Mu S. (2024). Identification and quantification of viable *Lacticaseibacillus rhamnosus* in probiotics using validated PMA-qPCR method. Front. Microbiol..

[48] Vasquez C., Leyton-Carcaman B., Cid-Alda F.P., Segovia I., Pinto F., Abanto M. (2023). Physical Pretreatments Applied in Three Commercial Kits for the Extraction of High-Quality DNA from Activated Sewage Sludge. Int. J. Mol. Sci..

[49] (2017). Method of PCR typing diagnosis for bacterial Part1: Mehtod of PCR detection for *Escherichia coli*.

[50] Chen S., Zhou Y., Chen Y., Gu J. (2018). fastp: An ultra-fast all-in-one FASTQ preprocessor. Bioinformatics.

[51] Li D., Liu C.-M., Luo R., Sadakane K., Lam T.-W. (2015). MEGAHIT: An ultra-fast single-node solution for large and complex metagenomics assembly via succinct *de Bruijn* graph. Bioinformatics.

[52] Hyatt D., Chen G.-L., LoCascio P.F., Land M.L., Larimer F.W., Hauser L.J. (2010). Prodigal: Prokaryotic gene recognition and translation initiation site identification. Bmc Bioinform..

[53] Noguchi H., Park J., Takagi T. (2006). MetaGene: Prokaryotic gene finding from environmental genome shotgun sequences. Nucleic Acids Res..

[54] Fu L., Niu B., Zhu Z., Wu S., Li W. (2012). CD-HIT: Accelerated for clustering the next-generation sequencing data. Bioinformatics.

[55] Li R., Li Y., Kristiansen K., Wang J. (2008). SOAP: Short oligonucleotide alignment program. Bioinformatics.

[56] Buchfink B., Xie C., Huson D.H. (2015). Fast and sensitive protein alignment using DIAMOND. Nat. Methods.

[57] Fu Y., Ye Z., Jia Y., Fan J., Hashmi M.Z., Shen C. (2020). An Optimized Method to Assess Viable *Escherichia coli* O157:H7 in Agricultural Soil Using Combined Propidium Monoazide Staining and Quantitative PCR. Front. Microbiol..

[58] Miwa T., Takimoto Y., Hatamoto M., Kuratate D., Watari T., Yamaguchi T. (2021). Role of live cell colonization in the biofilm formation process in membrane bioreactors treating actual sewage under low organic loading rate conditions. Appl. Microbiol. Biotechnol..

[59] Kang D., Zhang L., Yang S., Li J., Peng Y. (2023). Linking morphological features to anammox communities in a partial nitritation and anammox (PN/A) biofilm reactor. J. Environ. Manag..

[60] Liu M., Hata A., Katayama H., Kasuga I. (2020). Consecutive ultrafiltration and silica adsorption for recovery of extracellular antibiotic resistance genes from an urban river. Environ. Pollut..

[61] Caro D.M., Horstmann L., Ganzert L., Oses R., Friedl T., Wagner D. (2023). An improved method for intracellular DNA (iDNA) recovery from terrestrial environments. Microbiologyopen.

[62] Sivalingam P., Sabatino R., Sbaffi T., Fontaneto D., Corno G., Di Cesare A. (2023). Extracellular DNA includes an important fraction of high-risk antibiotic resistance genes in treated wastewaters. Environ. Pollut..

[63] Bairoliya S., Xiang J.K.Z., Cao B. (2022). Extracellular DNA in Environmental Samples: Occurrence, Extraction, Quantification, and Impact on Microbial Biodiversity Assessment. Appl. Environ. Microbiol..

[64] Grande R., Di Marcantonio M.C., Robuffo I., Pompilio A., Celia C., Di Marzio L., Paolino D., Codagnone M., Muraro R., Stoodley P. (2015). *Helicobacter pylori* ATCC 43629/NCTC 11639 Outer Membrane Vesicles (OMVs) from Biofilm and Planktonic Phase Associated with Extracellular DNA (eDNA). Front. Microbiol..

[65] Tang L., Schramm A., Neu T.R., Revsbech N.P., Meyer R.L. (2013). Extracellular DNA in adhesion and biofilm formation of four environmental isolates: A quantitative study. Fems Microbiol. Ecol..

[66] Ni J., Ji J., Li Y.-Y., Kubota K. (2023). Propidium monoazide—Polymerase chain reaction reveals viable microbial community shifts in anaerobic membrane bioreactors treating domestic sewage at low temperature. Bioresour. Technol..

[67] Codony F., Dinh-Thanh M., Agusti G. (2020). Key Factors for Removing Bias in Viability PCR-Based Methods: A Review. Curr. Microbiol..

[68] Van Holm W., Ghesquiere J., Boon N., Verspecht T., Bernaerts K., Zayed N., Chatzigiannidou I., Teughels W. (2021). A Viability Quantitative PCR Dilemma: Are Longer Amplicons Better?. Appl. Environ. Microbiol..

[69] Shi Q., Chen Z., Yan H., Xu M., Cao K.-F., Mao Y., Chen X., Hu H.-Y. (2023). Identification of significant live bacterial community shifts in different reclaimed waters during ozone and chlorine disinfection. Sci. Total Environ..

[70] Sun Y.-Q., Ge Y. (2023). Relic DNA effects on the estimates of bacterial community composition and taxa dynamics in soil. Appl. Microbiol. Biotechnol..

[71] Gensberger E.T., Polt M., Konrad-Köszler M., Kinner P., Sessitsch A., Kostić T. (2014). Evaluation of quantitative PCR combined with PMA treatment for molecular assessment of microbial water quality. Water Res..

[72] Kapoor V., Gupta I., Pasha A.B.M.T., Duc P. (2018). Real-Time Quantitative PCR Measurements of Fecal Indicator Bacteria and Human-Associated Source Tracking Markers in a Texas River following Hurricane Harvey. Environ. Sci. Technol. Lett..

