# Supplementary material for: Revealing the Viable Microbial Community of Biofilm in a Sewage Treatment System Using Propidium Monoazide Combined with Real-Time PCR and Metagenomics
Source: Microorganisms. 2024 Jul 23;12(8):1508. doi: 10.3390/microorganisms12081508 (PMC11356008; doi:10.3390/microorganisms12081508)
Supplement: Supplementary file 1 [file microorganisms-12-01508-s001.zip › microorganisms-3052226-supplementary.pdf]

Supplementary Information for

Revealing the Viable Microbial Community of Biofilm in Sewage Treatment System Using Propidium Monoazide Combined with Real-time PCR and Metagenomics

Jiayin Liang[1,2,+] Xiangqun Zheng[1,3,+] Tianyang Ning[1,2] Jiarui Wang[1,2] Xiaocheng Wei[1,2,*] Lu Tan[1,2,*] Feng Shen[1,2,*]

[1] Agro-Environmental Protection Institute, Ministry of Agriculture and Rural Affairs, No. 31 Fukang Road, Nankai District, Tianjin 300191, China

[2] Key Laboratory of Rural Toilet and Sewage Treatment Technology, Ministry of Agriculture and Rural Affairs, No. 31 Fukang Road, Nankai District, Tianjin 300191, China

[3]Institute of Environment and Sustainable Development in Agriculture, No.12 Zhongguancun South Street, Haidian District, Beijing 100081, China

[*]Corresponding Author: E-mail: weixiaocheng@caas.cn, E-mail: tanlu_004@126.com and E-mail: shenfeng@caas.cn

[+] These authors contributed equally to this work.

Texts and Figures chapters

Text S1 The reason for using *E. coli* as indicator strain

Text S2 Determination of concentration of *E. coli* suspension

Text S3 Specificity verification of primers

Figure S1. Gel electrophoresis diagram of *E. coli* primer specificity verification.

Figure S2. Multi-layer composite filler biological filter

Text S1 The reason for using *E. coli* as indicator strain

E. coli is often used as the indicator strain in the study of shielding efficiency of PMA in sewage or sludge against dead bacteria in sewage treatment system[1-3]. Therefore, the role of *E. coli* in this study was as an added indicator strain. For example, in a prior study which was published on Water Research in 2023[4], sewage and sludge treated with PMA successfully shielded more than 99% of dead bacteria by adding *E. coli* as an indicator strain. And Li et al. treated sewage and sludge using PMA concentration of 100 μM and a light exposure time of 4 min, and the results showed that matrices in sludge samples markedly reduced the effectiveness of PMA treatment by adding *E. coli* as an indicator strain[5]. The reasons for adding *E. coli* as the indicator strain is to determine the shielding efficiency of PMA on dead bacteria and the accuracy of qPCR results. As described by Liu et al., in the mixed bacteria system, the total inactivation of microorganisms requires a higher temperature and time. However, when the temperature is higher than 100 °C, some DNA will be degraded gradually. If the mixed bacteria are subjected to high temperature for a long time, the total DNA extraction efficiency will be reduced, making the results inaccurate[6,7] *E. coli* as one of the most common indicator strains. Its inactivation temperature is low, and it can be completely inactivated at 95 °C for 10 min. The use of *E. coli* as an indicator does not affect the subsequent DNA and qPCR analysis [8]. Therefore, adding *E. coli* as an indicator can more accurately evaluate the shielding efficiency of PMA against dead bacteria.

Text S2 Determination of concentration of *E. coli* suspension

The effectiveness of PMA treatment is closely associated with the concentration of bacterial suspension. Research by Lizheng Guo et al[9]. indicated that a bacterial suspension concentration of 1.0×10^8 CFU/mL facilitates bacterial and DNA extraction after centrifugation. Gradient dilution was performed on *E. coli* suspensions, and plates with colony counts ranging from 30 to 300 were selected for viable counting after plate culture. Based on calculations, a bacterial suspension concentration of 1.0×10^8 CFU/mL was chosen for subsequent experimental studies, and the suspension was subjected to $OD_{600\ nm}$ measurement. Plate counting and $OD_{600\ nm}$ measurement results indicated that at a concentration of 1.0×10^8 CFU/mL, the $OD_{600\ nm}$ value for *E. coli* suspension was 0.56. No colonies grew on plates after inactivation of *E. coli* suspension, indicating that exposure to boiling water for 20 min could completely thermally inactivate *E. coli*.

Text S3 Specificity verification of primers

PCR amplification and gel electrophoresis validation of *E. coli* primers were conducted, as indicated in Figure S2. The fragment size amplified by the *E. coli* primers matches that specified in the local standard DB21 "Method of PCR typing diagnosis for bacterial Part1: Mehtod of PCR detection for *Escherichia coli*" from Liaoning Province, which is 272 bp[10]. Only one band was produced, and no primer dimers were detected, indicating the specificity of the primer for subsequent PMA-qPCR experiments.

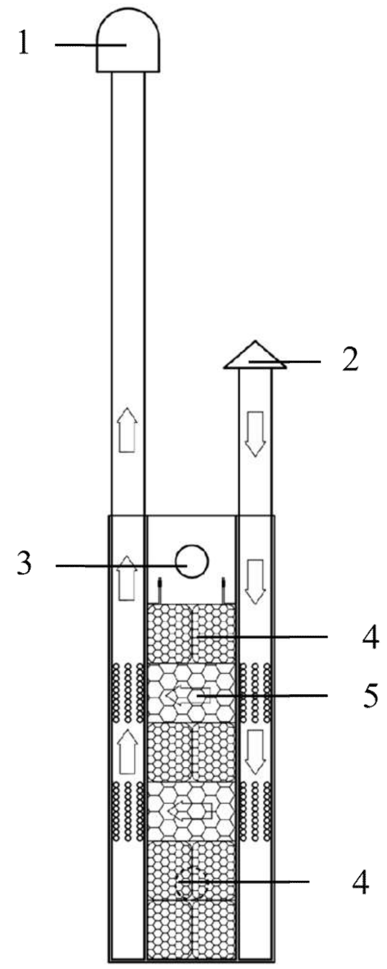

Figure S1. Multi-layer composite filler biological filter (1 powered hood, 2 air inlet, 3 water inlet, 4 water outlet, 5 filler one: zeolite, 6 filler two: polyurethane sponge. The direction pointed by the arrow is the direction of air flow)

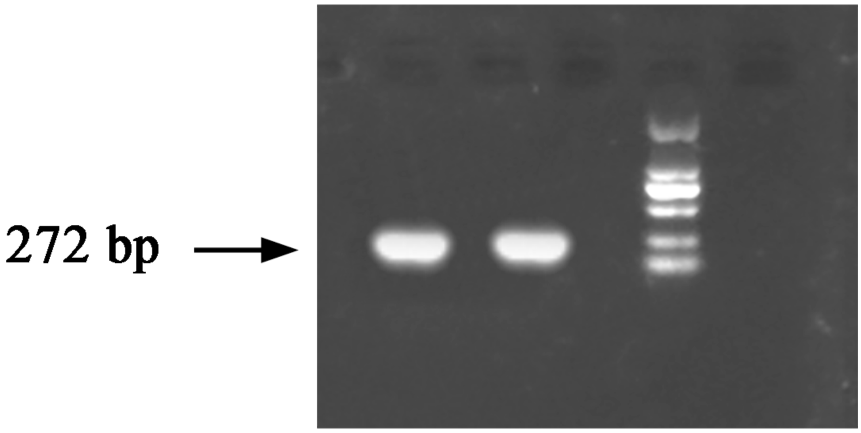

Figure S2. Gel electrophoresis diagram of *E. coli* primer specificity verification.

Reference

1. Zhang, X.; Lu, W.; Han, E.; Wang, S.; Shen, J. Hybrid Nanostructure-based Immunosensing for Electrochemical Assay of *Escherichia coli* as Indicator Bacteria Relevant to the Recycling of Urban Sludge. *Electrochimica Acta* **2014**, *141*, 384-390, doi:10.1016/j.electacta.2014.07.065.
2. Cooksey, E.M.; Singh, G.; Scott, L.C.; Aw, T.G. Detection of coliphages and human adenoviruses in a subtropical estuarine lake. *Science of the Total Environment* **2019**, *649*, 1514-1521, doi:10.1016/j.scitotenv.2018.08.322.
3. Kapoor, V.; Gupta, I.; Pasha, A.B.M.T.; Duc, P. Real-Time Quantitative PCR Measurements of Fecal Indicator Bacteria and Human-Associated Source Tracking Markers in a Texas River following Hurricane Harvey. *Environmental Science & Technology Letters* **2018**, *5*, 322-328, doi:10.1021/acs.estlett.8b00237.
4. Liu, W.; Xiang, P.; Ji, Y.; Chen, Z.; Lei, Z.; Huang, W.; Huang, W.; Liu, D. Response of viable bacteria to antibiotics in aerobic granular sludge: Resistance mechanisms and behaviors, bacterial communities, and driving factors. *Water Research* **2023**, *245*, doi:10.1016/j.watres.2023.120656.
5. Li, D.; Tong, T.; Zeng, S.; Lin, Y.; Wu, S.; He, M. Quantification of viable bacteria in wastewater treatment plants by using propidium monoazide combined with quantitative PCR (PMA-qPCR). *Journal of Environmental Sciences* **2014**, *26*, 299-306, doi:10.1016/s1001-0742(13)60425-8.
6. Liu, Y.; Schulze-Makuch, D.; de Vera, J.-P.; Cockell, C.; Leya, T.; Baque, M.; Walther-Antonio, M. The Development of an Effective Bacterial Single-Cell Lysis Method Suitable for Whole Genome Amplification in Microfluidic Platforms. *Micromachines* **2018**, *9*, doi:10.3390/mi9080367.
7. Karni, M.; Zidon, D.; Polak, P.; Zalevsky, Z.; Shefi, O. Thermal Degradation of DNA. *DNA and Cell Biology* **2013**, *32*, 298-301,

doi:10.1089/dna.2013.2056.
8. Macori, G.; McCarthy, S.C.; Burgess, C.M.; Fanning, S.; Duffy, G. Investigation of the Causes of Shigatoxigenic *Escherichia coli* PCR Positive and Culture Negative Samples. *Microorganisms* **2020**, *8*, doi:10.3390/microorganisms8040587.
9. Guo, L.; Ze, X.; Feng, H.; Liu, Y.; Ge, Y.; Zhao, X.; Song, C.; Jiao, Y.; Liu, J.; Mu, S.; Yao, S. Identification and quantification of viable *Lacticaseibacillus rhamnosus* in probiotics using validated PMA-qPCR method. Frontiers in Microbiology 2024, 15, doi:10.3389/fmicb.2024.1341884.
10. DB21/T 2734.1-2017. Method of PCR typing diagnosis for bacterial Part1: Mehtod of PCR detection for *Escherichia coli*. Standards Press of China, Liaoning.
